# Analysis of Benzo[a]pyrene in Vegetable Oils Using Molecularly Imprinted Solid Phase Extraction (MISPE) Coupled with Enzyme-Linked Immunosorbent Assay (ELISA)

**DOI:** 10.3390/s140609720

**Published:** 2014-05-30

**Authors:** Michael Pschenitza, Rudolf Hackenberg, Reinhard Niessner, Dietmar Knopp

**Affiliations:** 1 Institute of Hydrochemistry and Chemical Balneology, Chair for Analytical Chemistry, Technische Universität München, Marchioninistrasse 17, 81377 Munich, Germany; E-Mails: michael.pschenitza@mytum.de (M.P.); reinhard.niessner@ch.tum.de (R.N.); 2 Bundesamt für Verbraucherschutz und Lebensmittelsicherheit, Standort Marienfelde, Diedersdorfer Weg 1, 12277 Berlin, Germany; E-Mail: rudolf.hackenberg@bvl.bund.de

**Keywords:** polycyclic aromatic hydrocarbons, benzo[a]pyrene, vegetable oil, molecularly imprinted polymers, MISPE, ELISA, GC/MS

## Abstract

This paper describes the development of a molecularly imprinted polymer-based solid phase extraction (MISPE) method coupled with enzyme-linked immunosorbent assay (ELISA) for determination of the PAH benzo[a]pyrene (B[a]P) in vegetable oils. Different molecularly imprinted polymers (MIPs) were prepared using non-covalent 4-vinylpyridine/divinylbenzene co-polymerization at different ratios and dichloromethane as porogen. Imprinting was done with a template mixture of phenanthrene and pyrene yielding a broad-specific polymer for PAHs with a maximum binding capacity (*Q)* of ∼32 μg B[a]P per 50 mg of polymer. The vegetable oil/*n*-hexane mixture (1:1, (v/v)) was pre-extracted with acetonitrile, the solvent evaporated, the residue reconstituted in *n*-hexane and subjected to MISPE. The successive washing with *n*-hexane and isopropanol revealed most suitable to remove lipid matrix constituents. After elution of bound PAHs from MISPE column with dichloromethane, the solvent was evaporated, the residue reconstituted with dimethyl sulfoxide and diluted 100-fold with methanol/water (10:90, (v/v)) for analysis of B[a]P equivalents with an ELISA. The B[a]P recovery rates in spiked vegetable oil samples of different fatty acid composition were determined between 63% and 114%. The presence of multiple PAHs in the oil sample, because of MIP selectivity and cross-reactivity of the ELISA, could yield overestimated B[a]P values.

## 1. Introduction

Polycyclic aromatic hydrocarbons (PAHs) are a large group of substances consisting of molecules with fused aromatic ring systems. With incomplete combustion of organic compounds as their major source of formation, they are to be found ubiquitously in environmental samples of different kinds like soils and waters [1]. They have attracted considerable attention during the last decades because several PAHs have a high carcinogenic and mutagenic potential, e.g., causing cancer in lungs, skin and prostate [2,3] and are also considered endocrine disrupting compounds (EDC) [4]. The US Environmental Protection Agency (EPA) have identified 16 molecules with a wider range in molecular size as well as chemical and physiological properties, whereas in the EU the priority pollutants list contains the 15(+1) PAHs considered most harmful to human health [5,6]. Nevertheless, benzo[a]pyrene (B[a]P) is usually taken as marker substance for PAH exposure in general because of its extremely high carcinogenic potential [7]. Therefore, different limit values have been established for B[a]P in the EU in different matrices, e.g., air (2004/107/EC), drinking water (98/83/EC) and foodstuffs (835/2011).

For non-smokers PAHs in foodstuffs contribute most to PAH exposure [8]. Plants in particular tend to take up PAHs from the surrounding atmosphere and accumulate them on the surfaces of fruits, vegetables, seeds and corn. Contact with contaminated air can occur through industrial and traffic exhaust gases during plant growth, machinery exhaust during harvesting, storage or processing, as well as drying of plant material with combustion gases [9]. Due to their high hydrophobicity vegetable oils and fats show a good solubility for the lipophilic PAHs and therefore, PAH concentrations in the low μg/kg range can often be detected in edible oils and fats of different kinds [9–11]. To ensure efficient monitoring of PAH contents in these foodstuffs limit values have been set by the European Commission. For B[a]P a the maximum level was set to 2 μg/kg in edible oils and fats and also a sum value for B[a]P, benzo[a]anthracene, benzo[b]fluoranthene and chrysene of 10 μg/kg was established for most oils and fats (Commission Regulation 835/2011).

The analysis of PAHs at these low concentration levels is a major challenge to analytics, especially due to the laborious removal of the complex lipidic matrix for chromatographic detection. Two strategies are generally applied for PAH extraction from edible oils, the first of which is a combination of liquid-liquid extraction (LLE), typically using dimethyl sulfoxide (DMSO) followed by back-extraction with hexane with a solid phase extraction (SPE) step, for which normal-phase as well as reversed-phase (RP) solid phases can be applied [12–16]. More recently, improvements in SPE allow for omitting LLE and a direct SPE of the oil sample operating with sorbents like C_18_/Florisil^®^ or styrene/divinylbenzene copolymers [17,18]. For PAH quantitation chromatographic methods that are used for general PAH determination, like GC/MS or HPLC with fluorescence detection (FLD), are widely applied [19–22]. GC/MS as well as HPLC/FLD both require extensive sample preparation for the complex oil matrices typically resulting in time-consuming and expensive preparation procedures. In addition, both methods typically feature relatively long measuring times and require sophisticated systems for separation and detection.

With the detection of PAHs by immunoanalytical methods, however, low measurement costs can be combined with high sample throughput and usually also a short and simple sample preparation [23].

Performing the immunoassay directly in a neat hydrophobic solvent would be the method of choice. Over the last 30 years there were several studies published, mainly by Alexander Klibanov's group at MIT/Cambridge, on the action of enzymes in pure organic solvents [24,25]. Despite this, similar studies with antibodies are still rare. The first paper on the antibody-antigen binding in organic solvents of different hydrophobicity was published in 1989 [26]. With increasing hydrophobicity of the solvent a weaker protein-ligand interaction was observed for the model system 4-aminobiphenyl and its monoclonal antibody 2E11, although, the interaction was strong and specific also in non-aqueous media. Based on these findings, several advantages have been postulated, especially for the use of immunosensors in organic solvents [27,28]. Unfortunately, these optimistic expectations on their implementation in the analytical field were not fulfilled yet, *i.e.*, general investigations and reasonable results are still missing. So far, use of suitable organic solvent/aqueous media mixtures for running immunoassays with hydrophobic targets is the general accepted approach. This means that for each antigen-antibody pair the optimal solvent mixture has to be identified.

Nevertheless, for a sensitive detection of unpolar analytes in complex, hydrophobic matrices a thorough extraction/sample preparation and/or high dilution factors are needed [29–31]. Molecularly imprinted polymers (MIPs) show tailored binding sites for a selective analyte binding, thus making an efficient extraction and enrichment of analytes possible. This selectivity is combined with a high stability of the highly cross-linked organic polymers in terms of physical robustness, rigidity, resistance to elevated temperatures and pressures and inertness towards acids, bases, metal ions and organic solvents [32]. Several polymers, compositions and template molecules have been tested for PAH-MIP synthesis [33–41], of which, e.g., a 4-vinylpyridine/divinylbenzene copolymer showed good binding properties for B[a]P [42]. A drawback, however, is the use of the carcinogenic and costly B[a]P as a template molecule.

In this work, for the first time, we combined molecularly imprinted solid phase extraction (MISPE) and ELISA for analyzing B[a]P in edible oils. In addition, in contrast to earlier investigations, which focused on the preparation of MIP for B[a]P using the target analyte as template, we selected less toxic and cheap PAH template molecules for creating affine binding sites also to higher annelated compounds like B[a]P [42]. An extraction method was developed which permitted sensitive B[a]P analysis in the legally relevant concentration range using an ELISA procedure. Calibrations were made for different kinds of vegetable oil and recovery rates were tested using an olive oil reference material.

## 2. Experimental Section

### 2.1. Materials and Reagents

Flat-bottom, transparent 96-well polystyrene microplates were obtained from Greiner Bio-One (Frickenhausen, Germany). Spectroscopic measurements were carried out on a fluorimeter RF-5301PC (Shimadzu, Duisburg, Germany), a UV/Vis spectrometer Specord 250 Plus (Analytik Jena, Jena, Germany) and an ATR-FTIR-spectrometer Nicolet 6700 (Fisher Scientific GmbH, Schwerte, Germany). Horseradish peroxidase (HRP)-labelled anti-mouse IgG produced in horse was purchased from Axxora (Loerrach, Germany). Bovine serum albumin (BSA, fraction V, ∼99%) was purchased from Sigma-Aldrich (Taufkirchen, Germany). Hapten-protein conjugates were synthesized as published before [43]. The microtiter plates were washed automatically with a 96-channel plate washer (ELx405 Select) and the absorbance was measured with a microtiter plate reader (Synergy HT) both from Bio-Tek (Bad Friedrichshall, Germany).

Standard chemicals were obtained from Sigma-Aldrich. PAHs were purchased from PAH Research Institute (Greifenberg, Germany). 4-Vinylpyridine and divinylbenzene were distilled before use. Buffers and solutions were prepared freshly in ultrapure water which was generated using reverse osmosis with UV treatment (Milli-RO 5 Plus, Milli-Q185 Plus, Millipore, Eschborn, Germany). For the phosphate buffered saline (PBS, pH 7.6) 1.36 g potassium dihydrogen phosphate, 12.2 g dipotassium hydrogen phosphate, and 8.5 g sodium chloride in 1 L of water were used. Coating buffer (pH 9.6) consisted of 1.59 g disodium carbonate, 2.93 g sodium hydrogen carbonate, and 0.2 g sodium azide in 1 L of water. 1% Casein in PBS (w/v) was used as blocking reagent. The substrate buffer (pH 3.8) was prepared with 46.04 g potassium dihydrogen citrate and 0.10 g potassium sorbate in 1 L of water. The substrate solution consisted of 25 mL substrate buffer, 500 μL TMB stock solution (375 mg 3,3′,5,5′-tetramethylbenzidine in 30 mL of DMSO), and 100 μL 1% hydrogen peroxide. A 5% sulfuric acid solution was used to stop the enzymatic reaction. The washing buffer consisted of 42 mL washing buffer concentrate (8.17 g potassium dihydrogen phosphate, 73.16 g dipotassium hydrogen phosphate, 52.6 g sodium chloride, and 30 mL Tween 20 in 1 L of water) in 2.5 L of water.

### 2.2. MIP Preparation

With the exception of the template molecules used, the procedure for MIP preparation was carried out as published earlier [42]. In short, an overall template amount of 0.2 mmol (sum of pyrene and phenanthrene) was dissolved in dichloromethane (DCM, 2.0 mL) in a 4 mL screw-capped glass vial. As an optimal template ratio, 0.125 mmol of pyrene and 0.075 mmol of phenanthrene was used. After adding 4-vinylpyridine (105.1 mg, 1.0 mmol) the mixture was placed in the refrigerator at 4 °C for 30 min. Finally, divinylbenzene (651.0 mg, 5 mmol) and *N,N'*-azobisisobutyronitrile (AIBN, 30 mg) were added. The vials were placed on ice and deoxygenated with nitrogen for 5 min. Polymerization was carried out in a Biometra TB1 thermoblock (Goettingen, Germany) at 60 °C for 24 h. The resultant polymers were crushed, ground and sieved with 125 and 63 μm sieves. The polymer particles within the desired size range were washed with DCM while horizontally shaken for 24 h. DCM washing was repeated six times and after that the particles were sonicated in acetonitrile (MeCN) three times for 15 min each. After freeze drying the particles could be used for MISPE experiments.

### 2.3. Rebinding Experiments

In a 4 mL glass vial B[a]P in MeCN (0.5 mL) with known concentration was added to polymer particles (10 mg). The mixture was shaken horizontally for 20 h and then filtrated. B[a]P fluorescence in the filtrate was measured (λ_ex_ = 297 nm, λ_em_ = 405 nm). By subtracting the amount of B[a]P in the filtrate from the initial amount of B[a]P the maximum amount of B[a]P bound to the polymer (*Q*) could be calculated.

### 2.4. Extraction Method

Oil samples were pre-extracted by solvent extraction. Oil (1.5 mL) was placed in a glass centrifuge tube and diluted with *n*-hexane (1.5 mL). Dry Na_2_SO_4_ (2 g) was added and the mixture was extracted with MeCN (3.5 mL) by vortexing for 30 s and subsequent horizontal shaking for 5 min. After centrifugation (Hettich Universal 320R, Tuttlingen, Germany) for 5 min at 2450 g and 4 °C the MeCN phase was isolated and evaporated to dryness at 60 °C. The oily residue was reconstituted in *n*-hexane (1 mL) and subjected to MISPE.

For MISPE dry polymer (50 mg) was placed between two PTFE frits (porosity 10 μm; Merck Millipore, Darmstadt, Germany) in a 3 mL glass SPE cartridge. Before sample loading the column was washed with DCM and conditioned with *n*-hexane (2.5 mL). The LLE extract was applied to the column at a flow rate of about 0.1 mL · min^−1^. After drying the column with air it was washed with *n*-hexane (3 mL) and isopropanol (5 mL) successively. The column was eluted with DCM (5 mL), the solvent removed at 60 °C and the residue was reconstituted with dimethyl sulfoxide (DMSO, 0.75 mL). The extract in DMSO was then diluted 1:100 with water containing 10% methanol. This solution could then be measured using ELISA.

For calibration, the whole procedure was carried out with a blank oil sample and after dilution in water/methanol was spiked with B[a]P. A dilution series in the blank extract was carried out to obtain the B[a]P standard solutions.

**Figure 1.**
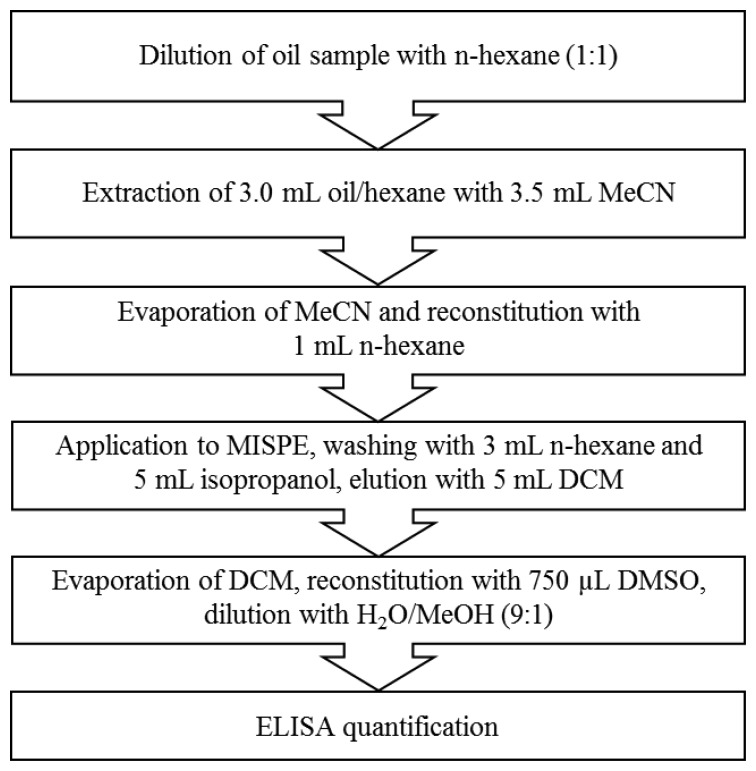
Schematic overview over the developed total procedure.

Microtiter plates were coated with fluoranthenyl-3-C4-BSA conjugate (1 mg/mL, dilution 1:15,000) dissolved in coating buffer (100 μL/well) and incubated at 4 °C overnight. The microtiter plates were washed automatically with washing buffer (300 μL/well) three times and incubated with blocking reagent (300 μL/well) for 1 h at room temperature with slight horizontal shaking at 300 rpm. After washing the microtiter plate the B[a]P calibration solution or the oil sample extract (50 μL/well) and the anti-B[a]P antibody diluted in PBS (50 μL/well, 1 mg/mL, dilution 1:20,000 in PBS) were added and the plate was shaken at 100 rpm for 1 h at room temperature. For establishing the calibration graph, B[a]P at concentrations of 0.001, 0.01, 0.05, 0.1, 0.5, 1, 10, 100 and 1000 μg/L in diluted oil extract was used. After another washing step, the plate was incubated with HRP-labelled anti-mouse antibody (dilution 1:5000 in PBS, 100 μL/well) at 100 rpm for 1 h at room temperature. After the last washing step substrate solution (100 μL/well) was added and incubated for 45 min at 100 rpm at room temperature. The colour development was stopped by adding 5% H_2_SO_4_ (100 μL/well). Finally, the absorption was measured with the plate reader at 450 nm. The schematic overview over the total procedure is shown in Figure 1.

### 2.5. Cross-Reactivity (CR) Determination

The CR of the anti-B[a]P monoclonal antibody was estimated with the 15(+1) EU PAHs using the indirect ELISA format accordingly. As coating conjugate B[a]P-10-C4-BSA was used (1 mg/mL, dilution 1:5000 in coating buffer). The PAH calibration solutions were prepared in methanol/water (10:90, (v/v)). The anti-B[a]P antibody was diluted 1:5000 in PBS. The percental CR was calculated by dividing the IC_50_ value of B[a]P by the IC_50_ of the tested compound and multiplied by 100.

### 2.6. GC/MS Measurements

A Thermo Trace GC from Thermo Fisher Scientific (Waltham, MA, USA) equipped with a Thermo DSQ mass spectrometer operating in electron ionization mode was used for GC determinations. Measurements were carried out on a 5 m × 0.25 mm i.d. (deactivated) precolumn and a 30 m × 0.25 mm i.d., 0.25 μm column (65% methyl-35%-phenylsilicone). Helium (99.999%, 1.0 mL/min) was used as carrier gas. The injection block temperature was kept at 250 °C and the oven temperature programme was changed as follows: 80 °C initial temperature, kept constant for 1.0 min, ramp to 230 °C at 20 °C/min, then ramp to 310 °C at 2 °C/min, kept constant for 15 min. Samples were injected in the splitless mode using a PAL GC autosampler (Axel Semrau GmbH & Co. KG, Sprockhövel, Germany) with an injection volume of 2 μL. The GC-MS interface was heated to 290 °C. The ion source temperature was kept at 230 °C and an ionization voltage of 70 eV was applied.

### 2.7. Selectivity of MIP

The selectivity of the MIP for the 15(+1) EU PAHs was calculated using results obtained with GC/MS. A solution of the PAHs in *n*-hexane (1 mL), each at a concentration of 75 μg/L, was applied to a MISPE column containing MIP (50 mg) and the flow-through was collected. The column was eluted with 5 mL DCM and after solvent evaporation the residue was reconstituted in *n*-hexane (1 mL). Peak areas obtained for the eluate were normalized using the values of the starting solution resulting and thus relative concentration values were calculated. Selectivity values were then calculated by dividing the concentration of each PAH by that of B[a]P and multiplying by 100.

## 3. Results and Discussion

### 3.1. Selection of Surrogate Template(s) for B(a)P

**Figure 2.**
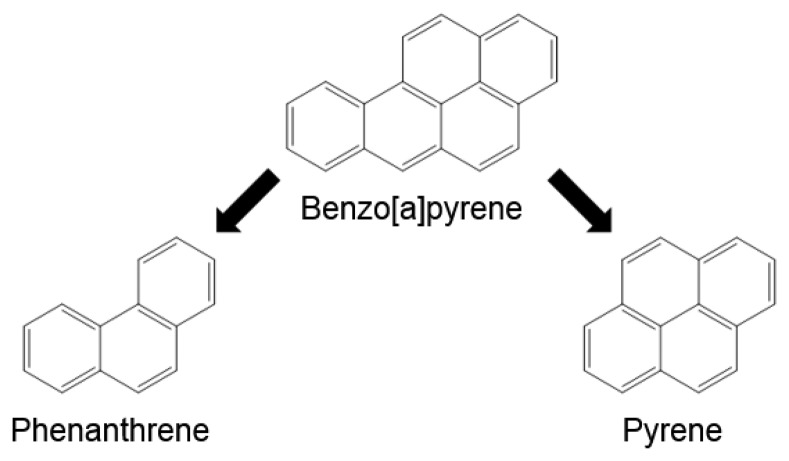
Creation of B[a]P binding sites using phenanthrene and pyrene as template mixtures.

For the synthesis of PAH binding MIPs usually high concentrations of template molecules, *i.e.*, of very toxic PAHs are required to provide a sufficient amount of binding sites on the polymer. As 4-vinylpyridine in combination with divinylbenzene has already been shown to yield a high binding MIP for B[a]P using the analyte itself as a template [42], we now tried to replace B[a]P in this procedure by less toxic and readily available PAHs without compromising binding capacity. The structural features of the B[a]P molecule are represented to a great extent also by pyrene lacking only the so called bay region formed between C_10_ and C_12_. To create binding sites targeting also this region pyrene was combined with phenanthrene (Figure 2) and different ratios were tested as template mixtures. As another advantage, cross reactivity of the used anti-B[a]P monoclonal antibody (22F12) against pyrene and phenanthrene is relatively low [23], so that analyte overestimation due to traces of template leaking from the polymer is minimized.

Different template compositions were tested ranging from 100% pyrene to 100% phenanthrene. The maximum binding capacities for each template composition are shown in Table 1. Obviously, binding capacity for B[a]P of MIPs is not significantly different relating to used template, *i.e.*, was almost the same for polymers prepared with pyrene, phenanthrene or mixtures of both. For comparison, using B[a]P as template the *Q*_MIP_/*Q*_NIP_ ratio was determined as 2.09 [42] which leads to the conclusion that replacing B[a]P by pyrene/phenanthrene mixtures, up to 97% of the binding capacity could be preserved. As maximum B[a]P binding seemed to be achieved between 0.10 and 0.15 mmol of pyrene, optimum concentrations of pyrene and phenanthrene of 0.125 mmol and 0.075 mmol, respectively, were applied in the following polymer syntheses.

### 3.2. Development of an Extraction Procedure Using Olive Oil

#### 3.2.1. B[a]P Binding of MIP Polymers in Different Solvents

To optimize applied sample volume and ensure quantitative B[a]P binding to MIP columns, B[a]P was loaded on MISPE columns in solvents of different polarity. B[a]P solutions were prepared in *n*-hexane, DMSO and MeCN at a concentration of 5 μg/L and applied in successive 1 mL aliquots to a MISPE column containing polymer (50 mg). The flow-through was collected and the B[a]P concentrations measured fluorimetrically.

Despite the very different polarities of *n*-hexane and MeCN, both solvents showed similar behavior regarding the binding of B[a]P to the MISPE column. After addition of 1 mL of the B[a]P solution more than 95% of the loaded B[a]P was bound on the column, even though small B[a]P concentrations (below LOQs) were detectable in the flow-through with no significant difference for *n*-hexane and MeCN. With DMSO, on the other hand, 63% of the initial amount of B[a]P could be detected in the flow-through after loading 1 mL of the solution on the column. With increasing sample volumes, steadily increasing B[a]P concentrations were detectable in the flow-through fractions for all solvents. Enrichment of B[a]P from higher sample volumes, therefore, was not considered conceivable. Because of its good solubility for oil matrix compounds and satisfactory binding of B[a]P to the MIP, *n*-hexane was chosen for the following experiments as sample solvent.

#### 3.2.2. Efficiency of Solvents for B[a]P Elution

**Figure 3.**
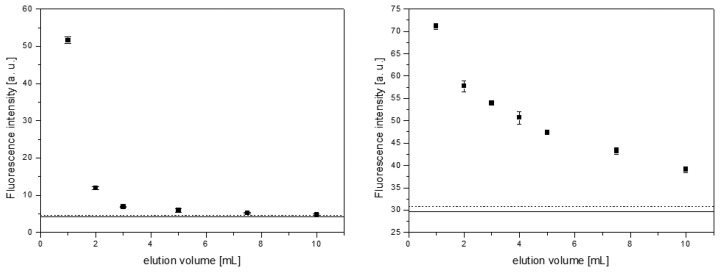
Fluorescence intensity of the different eluate fractions for DCM (**left**) and MeCN (**right**). The solid line represents the background intensity for each solvent and the dashed line the blank plus three times its standard deviation. All measurements were carried out in triplicates.

In earlier experiments dealing with MISPE of B[a]P from water and instant coffee samples, DCM could successfully be applied as eluent [42]. In the present study, DCM and MeCN were tested for B[a]P elution after previous application of unpolar samples. A solution of 10 μg/L B[a]P in *n*-hexane (1 mL) was loaded on a 50 mg MISPE column and B[a]P was eluted fractionately using up to 10 mL of DCM or MeCN, respectively. B[a]P concentrations in the different fractions were measured by fluorescence spectroscopy. Results are outlined in Figure 3.

For elution of B[a]P, DCM proved more practicable compared to MeCN. With the latter, B[a]P could still be detected in the eluate after addition of 10 mL of solvent, while with DCM B[a]P fluorescence intensity practically equaled the blank after application of 5 mL of solvent. The B[a]P elution, therefore, was carried out with 5 mL of DCM in all following experiments.

#### 3.2.3. Optimization of MISPE Washing Procedure

The efficiency of the washing step of the loaded column prior to elution is extremely important for an efficient separation of B[a]P from interfering matrix components, e.g., lipids in oil samples, which could hamper the immunoassay later on dramatically. The ability of different solvents to remove matrix components of olive oil was tested by loading a MIP column with 1 mL of a blank olive oil sample diluted with *n*-hexane (1:1) and washing the column with solvent in 1 mL-steps. An estimation of the matrix components was carried out by IR- and UV/Vis-spectroscopic measurements of the washing fractions. With IR-spectroscopy the concentration of fatty acids and their esters can be determined by the C–O double bond vibration (∼1750 cm^−1^), while the UV/Vis-spectra show the contents of plant pigments (chlorophylls and carotinoids). As solvents *n*-hexane, isopropanol, ethyl acetate and MeCN were tested. Results are summarized in Figures S1–S3 in the Supplementary Information. They clearly indicated that MeCN was the only solvent with an insufficient solubility for lipidic matrix compounds and was therefore not considered further as a washing solvent. The remaining three solvents showed almost equal properties regarding their solubility for lipidic matter. After addition of 3 mL of solvent, the IR-spectra showed a complete disappearance of the C–O band and also the typical absorption bands for olive oil in the UV/Vis-spectra (400–500 nm and 600–700 nm) had practically vanished.

**Figure 4.**
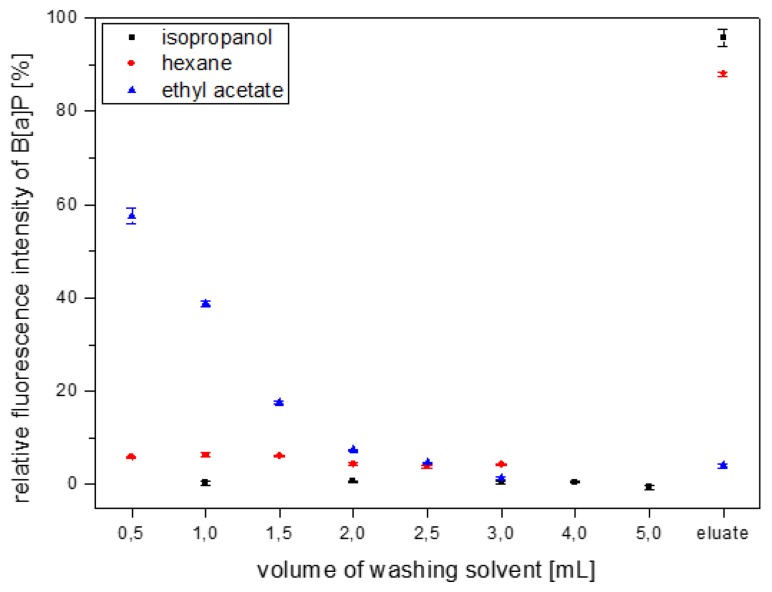
Investigation of isopropanol, *n*-hexane and ethyl acetate as MISPE washing solvent assessed by B[a]P leakage.

Thus, *n*-hexane, isopropanol and ethyl acetate were tested for their ability to retain bound B[a]P on the MIP column during the washing step. In detail, B[a]P dissolved in *n*-hexane was loaded on the column, then the column was washed fractionately with one of the three solvents and finally, B[a]P was eluted using 5 mL DCM. The B[a]P concentrations in the washing fractions as well as the eluates were determined by fluorescence spectroscopy (Figure 4).

Ethyl acetate removed B[a]P completely after addition of 3 mL of solvent, so that almost no B[a]P could be detected in the eluate. It therefore was discarded as a washing solvent. In the isopropanol washing fractions no B[a]P could be detected and recovery in the elution after a total of 5 mL isopropanol was about 95%. With *n*-hexane, a slight B[a]P leaking could be seen in all fractions. Nevertheless, B[a]P recovery after addition of 3 mL of *n*-hexane was still considered satisfactory. To ensure maximum removal of matrix components, a combined washing with 3 mL *n*-hexane followed by 5 mL isopropanol was considered most suitable.

### 3.3. Extraction of B[a]P from Edible Oils for ELISA Quantification

#### 3.3.1. Optimization of Assay Sensitivity

As the elution solvent DCM is incompatible with the immunoassay due to its low polarity, it has to be evaporated and the residue reconstituted in a more polar solvent, which can finally be diluted in water or water/alcohol mixtures. The solubility of B[a]P in pure water is estimated about 4 μg/L [44]. Concluding, to ensure B[a]P solubility also in higher concentrations, at the same time maintaining high antibody affinity, mixtures of water containing 10% methanol had proved useful [23,43]. To fully solubilize the elution residue, however, small volumes of a less polar, but still water miscible solvent had to be applied, which could then be diluted with water/methanol.

Using MeCN as reconstitution solvent, ELISA calibration curves could not be obtained in a reproducible way with sufficient assay sensitivity. DMSO, on the other hand, gave reproducible calibrations, so the reconstitution volume as well as the dilution factor in water/methanol (90:10, (v/v)) were optimized to obtain high assay sensitivity and to enable detection of legally relevant B[a]P concentrations. As a measure for assay sensitivity, the IC_50_ values of the sigmoidal calibration curves were compared. A DMSO volume of 1.5 mL and its fiftyfold dilution in water/methanol gave the lowest IC_50_ value, although flawed by a high uncertainty of the value (Table 2). Lower errors could be obtained by higher dilution factors of the DMSO in water/methanol, but also a decrease in assay sensitivity was observed. As optimum values 750 μL of DSMO and a dilution factor of 1:100 in water/methanol were chosen.

The substitution of the B[a]P-BSA coating conjugate by a fluoranthenyl-BSA conjugate, revealed a further decrease of the IC_50_ value, as has already been showed before [45]. A decrease of the LOD below the legal limit value of 2 μg/kg for edible oils could be achieved by titration of anti-B[a]P antibody and coating conjugate to obtain the assay conditions providing the highest assay sensitivity (Table 3). As a minimum IC_50_ value and also a minimum lower limit of the working range were obtained with an antibody dilution of 1:20,000 and a dilution of the coating conjugate of 1:15,000, these conditions were chosen for the following experiments.

In order to determine the precision of the ELISA calibration three separate curves were established with the same extract. The IC_50_ values were averaged to estimate the standard deviation of the curves yielding a value of 7.58 ± 0.79 μg/kg. In addition, deviations between different extractions were determined by extracting a blank oil sample three times and establish calibration curves with each diluted extract. For the three curves, an average IC_50_ value of 8.95 ± 0.96 μg/kg was obtained. With both standard deviations being roughly about 10%, which is a typical value regard repeatability of immunoassays, the assay and the extraction were considered repeatable.

#### 3.3.2. Assay Calibration Using Different Vegetable Oils

Vegetable oils mainly consist of glycerol triesters of a variety of fatty acids. The chemical properties of these fatty acids depend to a great extent on the number and position of C=C double bonds within the hydrocarbon chain. For the development of the MISPE method for B[a]P from edible oils an olive oil was used as a model matrix because of its intermediate matrix composition with mono-unsaturated fatty acids accounting for about 75% of the total fatty acid content [46]. In order to test the influence of different matrices, *i.e.*, fatty acid compositions, a series of common vegetable oils was extracted and calibration curves were established with the reconstituted and diluted extracts.

As can be seen from Table 4, the parameters of the calibration curves are very similar with different oils, so the effect of the remaining oil constituents on the immunoassay performance seems to be negligible. LOD values were below 2 μg/kg for all tested oil samples and thus, B[a]P detection above the limit value should be possible independent of the type of oil.

#### 3.3.3. B[a]P Detection in Different Vegetable Oil Samples

To demonstrate the potential of the described method for B[a]P detection in edible oils, three oil samples with different fatty acid composition (olive oil, sunflower oil, linseed oil) were spiked with B[a]P in different concentrations above the set limit value and B[a]P concentrations measured for determination of recovery rates. B[a]P recovery ranged from 63% to 114% (Table 5). For olive and linseed oil, underestimations of the spiked concentrations were observed in all samples, while for sunflower oil recoveries were generally higher.

For further validation, a reference olive oil sample was obtained from the *Bundesamt für Verbraucherschutz und Lebensmittelsicherheit, Berlin*. The concentrations of the 15(+1) EU priority PAHs had been determined by GC/MS. The sample was subjected to the developed MISPE and B[a]P equivalent concentration measured using ELISA (*Note: Because the used anti-B[a]P antibody is cross-reactive with other PAHs, the measured ELISA signal has to be considered a sum signal, designated as B[a]P equivalents for PAHs existing in the sample* [23]). In detail, each individual PAH concentration was multiplied with the percentage MIP selectivity and antibody cross-reactivity compared to B[a]P (Table 6). As expected, an overestimation of the B[a]P concentration measured by MISPE-ELISA compared to GC/MS was observed, *i.e.*, the ELISA value of 5.4 μg/kg was higher by a factor of 2.1. However, the theoretical B[a]P equivalent concentration of 11.07 μg/kg which was calculated based on the cross-reactivity values of the anti-B[a]P antibody against all of the 15(+1) PAHs which were present in the oil sample was even higher. This value was underestimated clearly by the MISPE-ELISA method by a factor of about two. The finding, however, is not surprising and can be explained by the competition of the different PAHs for the limited antibody binding sites of the ELISA. Only those compounds which exhibit highest binding affinity (cross-reactivity) will contribute to the sum signal predominantly. Concluding, the determined MISPE-ELISA values, owing to the broad-specific retention of PAHs on the MISPE column and the CR-pattern of B[a]P-ELISA, represent a sum signal of relevant PAHs contained in the oil samples. If the obtained B[a]P equivalent concentration is below the limit value of 2 μg/kg set for B[a]P, the oil sample can be considered as clearly negative, *i.e.*, B[a]P concentration is below the MRL value. Otherwise, higher concentrations do not allow deduction with regard to B[a]P content. In such cases, further chromatographic analyses are recommended.

## 4. Conclusions

For the first time, molecularly imprinted solid phase extraction (MISPE) and ELISA was combined for analyzing B[a]P in edible oils. In contrast to earlier investigations, which focused on the preparation of MIP for B[a]P using the target analyte as template, less toxic PAH template molecules pyrene and phenanthrene were used for creating affine binding sites. The binding of B[a]P from different solvents was tested using *n*-hexane, DMSO and MeCN. It was found, that about 95% of B[a]P were bound to the column from *n*-hexane and MeCN, while with DMSO more than 60% of the loaded B[a]P were detected in the flow through. For elution, however, DCM proved most useful and complete elution of B[a]P from the column could be achieved with 5 mL of solvent. An efficient washing procedure for MISPE columns after addition of edible oil extracts could be established using 3 mL *n*-hexane and 5 mL isopropanol successively. It could be demonstrated, that both solvents ensure high efficiency of matrix removal while retaining B[a]P on the MISPE column. For detection of B[a]P, MISPE was combined with an immunoanalytical detection using an anti-B[a]P ELISA. For that, the residue remaining after evaporation of the elution solvent DCM was taken up in DMSO and diluted in water containing 10% methanol. DMSO volume as well as the dilution factor in water/methanol were optimized to ensure detection within the legally relevant concentration range (2 μg/kg for B[a]P in edible oils). Calibration of the ELISA was carried out in extracts of different kinds of oil samples with varying fatty acid compositions. The B[a]P recovery rates in spiked oil samples were determined between 63% and 114% for olive oil, sunflower oil and linseed oil. The analysis of an olive oil sample which contained all of the 15(+1) PAHs from EU priority list and was determined in parallel by GC/MS revealed an overestimation of B[a]P concentration by a factor of about 2. This was clearly shown to be caused by the broad selectivity of the as-prepared MIP polymer and cross-reactivity pattern of the used anti-B[a]P-antibody. Regardless of this limitation, any B[a]P concentration below the set MRL value of 2 μg/kg as obtained by the described MISPE-ELISA would clearly indicate an acceptable quality of the edible oil. Disregard the obtained promising results, the method is still at an early stage, *i.e.*, is a proof of concept rather than a fully validated method which has to comprise very detailed quality assurance/quality control (QA/OC) procedures [47].

## Supplementary Material



## Figures and Tables

**Table 1.** Comparison of maximum binding capacities *Q* for B[a]P of MIPs with different template compositions and a non-imprinted polymer (NIP).

Template Concentration [mmol]		*Q* [μg/g]	*Q*(MIP)/Q(NIP)

Pyrene	Phenanthrene		
0	0	15.9	-	
0	0.20	29.9	1.88	
0.05	0.15	30.6	1.93	
0.10	0.10	32.1	2.02	
0.15	0.05	31.9	2.01	
0.20	0	31.2	1.97	

**Table 2.** Influence of applied solvent volume for eluate residue reconstitution, dilution factor in water/methanol (90:10, (v/v)) and overall dilution factor on ELISA sensitivity as estimated by the IC_50_ value of ELISA calibration curves (n = 8, m = 3). The sample was olive oil.

DMSO Volume [μL]	Dilution in Water/MeOH (90:10, (v/v))	Overall Dilution Factor	Calibration Possible?	IC_50_ Value [μg/kg]
375	1:100	1:25	no	
750	1:50	1:25	no	
1500	1:25	1:25	no	
500	1:100	1:33.3	yes	43.0 ± 19.4
750	1:75	1:37.5	yes	48.9 ± 26.1
375	1:200	1:50	yes	36.9 ± 0.6
750	1:100	1:50	yes	36.5 ± 6.2
1500	1:50	1:50	yes	24.4 ± 10.6
375	1:400	1:100	yes	50.3 ± 10.2
750	1:200	1:100	yes	46.5 ± 11.0
1500	1:100	1:100	yes	59.1 ± 4.1

**Table 3.** Variation of ELISA conditions for further optimization of assay sensitivity (n = 8, m = 3).

Antibody Dilution	Coating Antigen Dilution	IC_50_ [μg/kg]	Working Range [μg/kg] (20%–80% of Max. Signal)	LOD [μg/kg]
1:20,000	1:5,000	8.10	2.56–25.66	1.27
1:20,000	1:10,000	8.25	2.35–29.15	2.34
1:20,000	1:15,000	5.45	2.06–14.45	1.79
1:40,000	1:5,000	52.05	4.78–567.38	4.24

**Table 4.** ELISA calibration parameters, IC_50_ value, limit of detection (LOD), and linear working range (WR), with vegetable oils of different fatty acid (FA) composition (n = 8, m = 3). FA values were taken from [46].

Oil	IC50 [μg/kg]	LOD [μg/kg]	WR [μg/kg]	Saturated FA [wt%]	Mono-Unsaturated FA [wt%]	Poly-Unsaturated FA [wt%]
coconut oil	7.87	0.61	2.44–25.43	90	7	2
palm oil	10.50	1.35	2.95–37.38	46	39	11
olive oil 1	10.66	0.65	3.45–32.88	12	78	8
olive oil 2	10.18	1.39	2.68–38.68			
peanut oil	9.33	1.51	2.74–31.69	7–15	46–71	14–35
sunflower oil	7.68	1.59	2.01–29.36	11	20	60
grape seed oil	7.32	1.37	2.11–25.44	11	18	71
linseed oil *	9.30	0.73	2.06–41.95	9	17	74

* containing high amounts of tri-unsaturated fatty acids.

**Table 5.** B[a]P recovery rates in spiked vegetable oil samples.

Edible Oil	Spiked B[a]P Concentration [μg/kg]	Measured B[a]P Concentration [μg/kg]	Recovery Rate [%]
olive oil	2.5	2.47 ± 0.10	99
	5	3.24 ± 0.19	65

sunflower oil	2.5	2.86 ± 0.30	114
	6	4.70 ± 0.29	78

linseed oil	2.5	2.36 ± 0.09	94
	6	3.75 ± 0.12	63

**Table 6.** Calculation of B[a]P equivalents of the 15(+1) EU PAHs based on MIP selectivity and anti-B[a]P antibody cross-reactivity.

PAH	c [μg/kg]	MIP Selectivity [%]	Cross-Reactivity [%]	B[a]P Equivalent [μg/kg]
Benzo[a]pyrene	2.56	100	100	2.56
Benzo[c]fluorene	4.68	43	6	0.12
Benzo[a]anthracene	1.84	71	13	0.17
Chrysene	4.02	80	77	2.47
Benzo[b]fluoranthene	4.32	87	24	0.90
Benzo[j]fluoranthene	4.65	94	45	1.96
Benzo[k]fluoranthene	1.96	86	5	0.08
Cyclopenta[cd]pyrene	3.66	75	3	0.09
Dibenzo[ah]anthracene	1.10	104	<0.1	0.00
Benzo[ghi]perylene	3.60	96	1	0.03
Dibenzo[ae]pyrene	2.05	105	<0.1	0.00
Dibenzo[ah]pyrene	2.34	111	3	0.08
Dibenzo[ai]pyrene	3.09	116	6	0.21
Dibenzo[al]pyrene	1.90	84	<0.1	0.00
Indeno[1,2,3-cd]pyrene	5.17	99	45	2.31
5-Methylchrysene	2.62	66	5	0.08

				sum: 11.07
