# Supplementary material for: Analysis of Benzo[a]pyrene in Vegetable Oils Using Molecularly Imprinted Solid Phase Extraction (MISPE) Coupled with Enzyme-Linked Immunosorbent Assay (ELISA)
Source: Sensors (Basel). 2014 May 30;14(6):9720–37. doi: 10.3390/s140609720 (PMC4118365; doi:10.3390/s140609720)
Supplement: Supplementary file 1 [file sensors-14-09720-s001.pdf]

OPEN ACCESS

sensors

ISSN 1424-8220

www.mdpi.com/journal/sensors

Supplementary Information

Analysis of Benzo[a]pyrene in Vegetable Oils Using Molecularly Imprinted Solid Phase Extraction (MISPE) Coupled with Enzyme-Linked Immunosorbent Assay (ELISA)

Michael Pschenitza [1], Rudolf Hackenberg [2], Reinhard Niessner [1] and Dietmar Knopp [1,*]

[1] Institute of Hydrochemistry and Chemical Balneology, Chair for Analytical Chemistry, Technische Universität München, Marchioninistrasse 17, 81377 Munich, Germany; E-Mails: michael.pschenitza@mytum.de (M.P.); reinhard.niessner@ch.tum.de (R.N.)

[2] Bundesamt für Verbraucherschutz und Lebensmittelsicherheit, Standort Marienfelde, Diedersdorfer Weg 1, 12277 Berlin, Germany; E-Mail: rudolf.hackenberg@bvl.bund.de

* Author to whom correspondence should be addressed; E-Mail: dietmar.knopp@ch.tum.de; Tel.: +49-89-2180-78252; Fax: +49-89-2180-78255.

Figure S1. Infrared spectra of sample (olive oil, diluted 1:1 with *n*-hexane) and respective washing fractions for estimation of fatty acid concentrations. Solvents used for washing are *n*-hexane, isopropanol, ethyl acetate and acetonitrile. For ethyl acetate, washing efficiency was estimated by alkyl bands (2750–3000 cm^{-1}, see Figure S2), for all other solvents the C–O peak at about 1750 cm^{-1} was used.

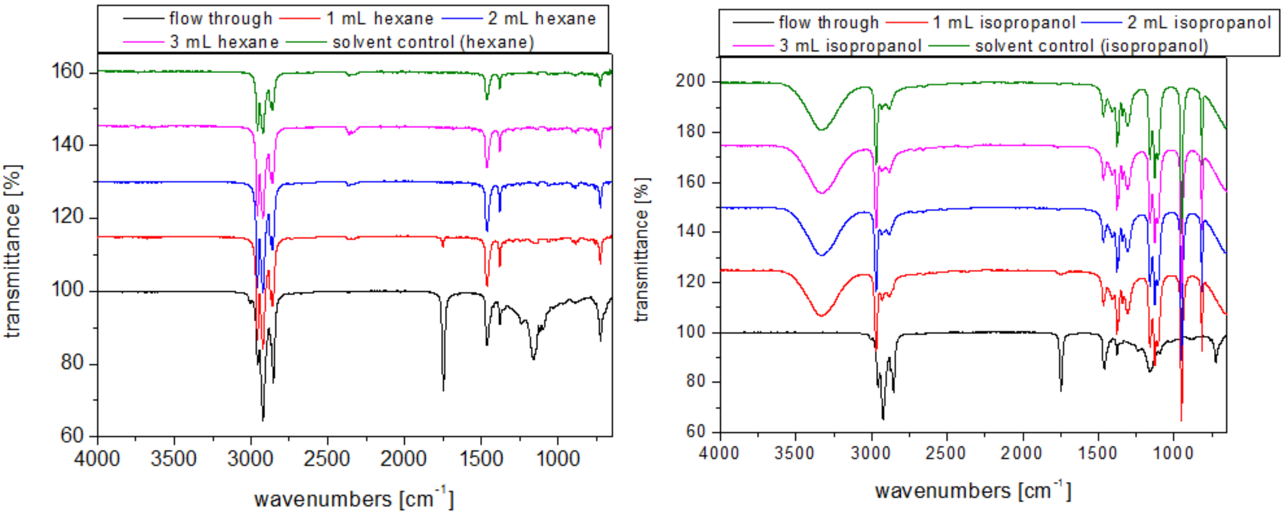

Figure S1. *Cont.*

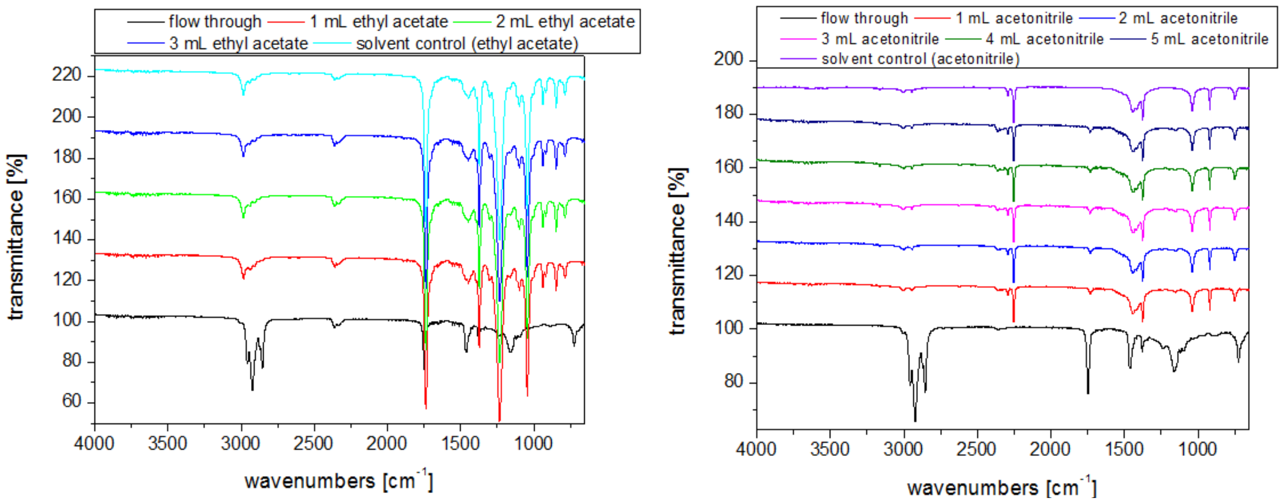

Figure S2. Infrared spectra of sample (olive oil, diluted 1:1 with *n*-hexane) and washing fractions for ethyl acetate enlarged for alkyl vibrations.

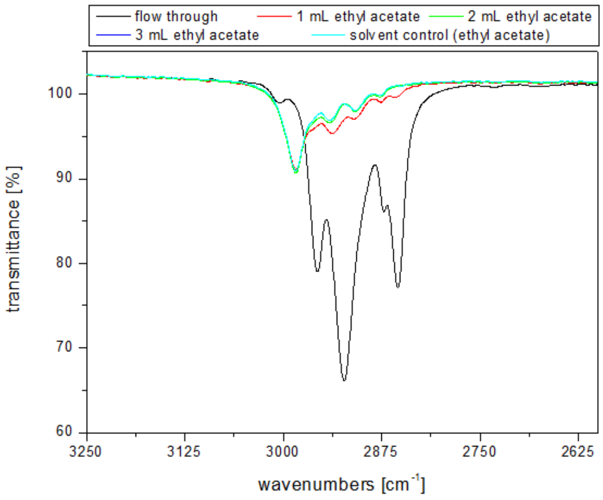

Figure S3. UV/V is spectra of sample (olive oil, diluted 1:1 with *n*-hexane) and washing fractions for estimation of pigment content (400–500 nm and 600–700 nm). Solvents were the same as above.

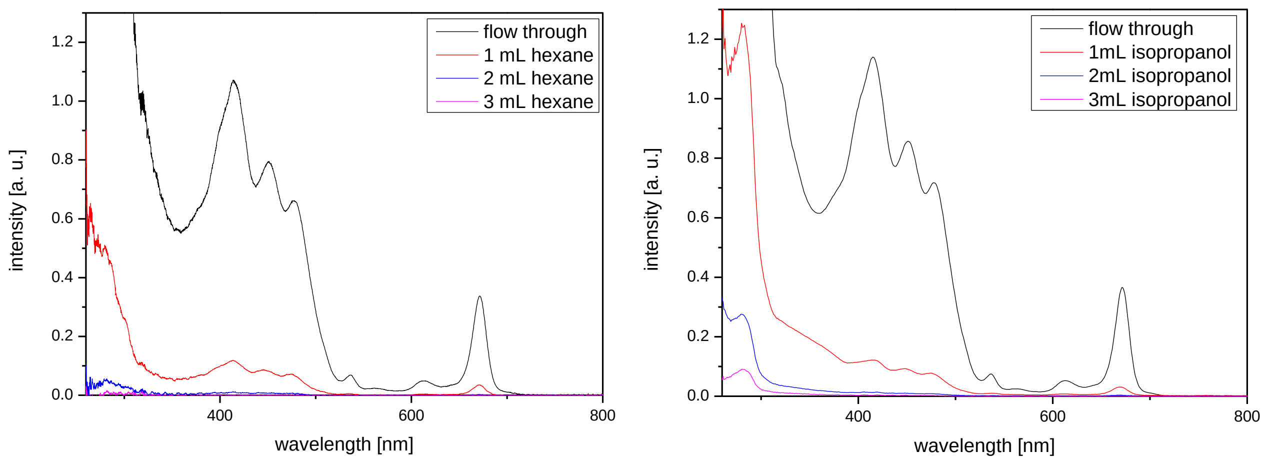

Figure S3. *Cont.*

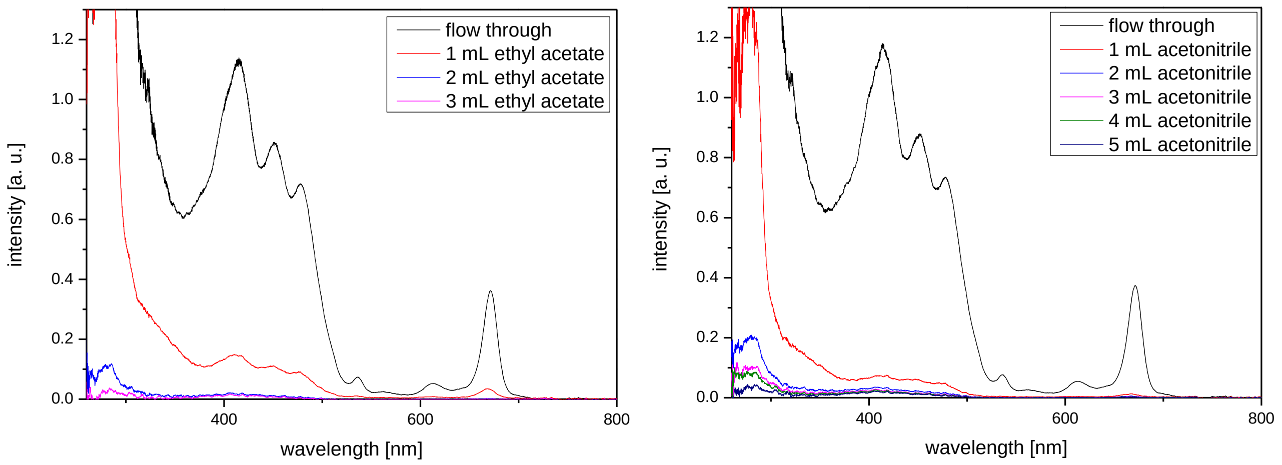

© 2014 by the authors; licensee MDPI, Basel, Switzerland. This article is an open access article distributed under the terms and conditions of the Creative Commons Attribution license (http://creativecommons.org/licenses/by/3.0/).
